# Cochleovestibular findings linked to COVID-19: A scoping review for clinical care planning in South Africa

**DOI:** 10.4102/sajcd.v69i2.899

**Published:** 2022-08-12

**Authors:** Katijah Khoza-Shangase

**Affiliations:** 1 Department of Audiology, Faculty of Humanities, University of the Witwatersrand, Johannesburg, South Africa

**Keywords:** audiology, cochleovestibular, clinical, COVID-19, hearing loss, planning, South Africa, steroids, tinnitus, vertigo

## Abstract

**Background:**

On 30 January 2020, the World Health Organization (WHO) officially declared an outbreak of the coronavirus disease 2019 (COVID-19) to be a global health emergency. Research has focused on the impact and response to life-threatening symptoms of COVID-19 across the lifespan; however, there is a need to investigate the effects of COVID-19 on the cochleovestibular system, as viral infections are known to impact this system. This is particularly important for contexts where resources are limited and prioritisation of resources requires strong risk versus benefit evaluations.

**Objective:**

Therefore, the purpose of this scoping review was to investigate published evidence on the impact of COVID-19 on the cochleovestibular system across the lifespan in order to allow for strategic clinical care planning in South Africa, where capacity versus demand challenges exist.

**Methods:**

Electronic bibliographic databases such as CINAHL, EBSCOHost, MEDLINE, ProQuest, PubMed, Scopus and ScienceDirect were searched for peer-reviewed publications between January 2020 and January 2022. These had to be published in English and related to the impact of COVID-19 on the cochleovestibular system, where the question was: ‘what evidence has been published on the impact of COVID-19 on the cochleovestibular system?’ Review selection and characterisation was performed by the researcher with an independent review by a colleague using pretested forms.

**Results:**

Of a total of 24 studies that met the inclusion criteria, the current scoping review revealed limited conclusive published evidence linking COVID-19 to permanent hearing function symptoms. Current evidence supports the possibility of COVID-19, similar to other viral infections in adults, impacting the cochleovestibular system and causing tinnitus, vertigo and sudden sensorineural hearing loss (SSNHL), with the symptoms being generally temporary and resolving either partially or completely following therapy with steroids, with very inconclusive findings in the paediatric population.

**Conclusion:**

These findings raise global implications for properly designed studies, which include longitudinal follow-up of cases across the lifespan, examining this link with some focus on establishing the pathophysiologic mechanisms at play as well. In the meanwhile, current findings raise the value of polymerase chain reaction (PCR) testing for all patients presenting with unexplained cochleovestibular symptoms during the pandemic, as these may be the only presenting symptoms indicating COVID-19, thus requiring careful treatment and management.

## Introduction

Since the advent of coronavirus disease 2019 (COVID-19), which was first reported in Wuhan, China, the World Health Organization (WHO) officially declared this novel coronavirus outbreak an international global public health emergency on 30 January 2020 and finally a pandemic on 11 March 2020 (WHO, 2020a, 2020b). As of 21 June 2022, the total global number of confirmed cases has escalated to 540 million with over 6.32 million deaths, with South Africa registering a cumulative total of 3.99 million cases and 102 000 total official deaths (WHO, 2022). These numbers were regardless of all the global efforts to contain the virus (Hui et al., 2020; WHO, 2022). Thus, prevention and control of the COVID-19 infection, including during research, as well as provision of clinical care and training, is a key strategy to save lives and livelihoods, while safeguarding the safety of healthcare professionals and patients (Beach et al., 2020; Khoza-Shangase, Moroe, & Neille, 2021; Wieten, Burgart & Cho, 2020; WHO, 2022).

During the current COVID-19 pandemic, the wearing of face coverings globally is one of the key prevention strategies recommended and in some countries mandated. Community-wide face covering wearing, particularly in public areas, as is the requirement in South Africa (Balkaran & Lukman, 2021), is aimed at decreasing possible symptomatic or pre-symptomatic transmission of COVID-19 to others, along with adhering to physical distancing and handwashing or sanitising (Ten Hulzen & Fabry, 2020). Logically, wearing of face coverings impacts aspects of communication such as visual cues (lip reading, facial expressions) during communication – particularly for those who rely on this compensatory strategy because of a hearing impairment (Chodosh, Weinstein, & Blustein 2020; Homans & Vroegop, 2021; Kataoka, Maeda, Sugaya, Omichi, & Kariya, 2021; Maru, Stancel-Lewis, Easton, & Leverton, 2021).

Evidence suggests that universal wearing of face coverings and adherence to physical distancing during COVID-19 has a serious negative impact on communication for the hearing impaired (Naylor, Burke, & Holman, 2020; Saunders, Jackson, & Visram, 2021; Ten Hulzen & Fabry, 2020). Goldin, Weinstein and Shiman (2020) found that masks lead to an attenuation of the speaker’s voice in the 2000 Hz – 7000 Hz frequency range because of a low-pass filter effect. Furthermore, a reduction in sound intensity of 3 dB to 4 dB for medical mask to approximately 12 dB for the N95 mask (respirator or FFP) was found. This, coupled with physical distancing, exacerbates the deleterious impact of COVID-19 on communication, particularly for the hearing impaired (Ten Hulzen & Fabry, 2020). If COVID-19 directly impacts hearing function, a real hearing impairment may be overlooked by the patient and their family if these documented effects of mask wearing and physical distancing are taken into consideration.

In a recent online survey within the United Kingdom, where the goal was to explore experiences of interactions where face masks were worn and how these masks affected communication, Saunders et al. (2021, p. 495) found that face masks had a negative impact on ‘hearing, understanding, engagement and feelings of connection with the speaker’ with this impact being more pronounced in hearing-impaired individuals. Similar findings were reported by Naylor et al. (2020). Researchers such as Gallus et al. (2021) and Almufarrij and Munro (2021) reported on dizziness, rotatory vertigo, dynamic imbalance and static imbalance that is transitory in nature, without any solid evidence of clinically significant permanent vestibular damage post COVID-19 infection. This evidence has important implications for early identification of real cochleovestibular symptoms that might be linked to COVID-19 so that they are not lost in these ‘perceived’ temporary subjective impacts, hence the importance of careful documentation of the possible impact of COVID-19 on the cochleovestibular system. Vestibular symptoms may be more obvious and easier to identify and report, particularly in the absence of accompanying major COVID-19 symptoms, but hearing symptoms might remain hidden.

Viral infections such as cytomegalovirus, measles, herpes simplex virus, HIV, rubella and others have been associated with hearing loss (Assuiti et al., 2013; Caroça et al., 2017; Cohen, Durstenfeld, & Roehm, 2014; Dunmade, Segun-Busari, Olajide, & Ologe, 2007; Goderis et al., 2014; Khoza-Shangase, 2010; Khoza-Shangase & Anastasiou, 2020). Cohen et al. (2014) reported that viral infections impact cochleovestibular function by either directly causing damage to inner ear structures, such as the inner hair cells and the organ of Corti, or by stimulating an inflammatory response on the auditory system. It is thus not surprising to anticipate that COVID-19, as a viral infection, might also impact hearing and balance function, although cochleovestibular symptoms have not been reported as part of the core symptoms of the disease. Mustafa (2020) argued that COVID-19, as a new pandemic that is viral in nature, requires scrutiny insofar as its possible impact on the audiological system.

The commonly documented clinical features of COVID-19 include dry cough, sore throat, headache, fever, fatigue, loss of taste, loss of sense of smell and shortness of breath (Alimohamadi, Sepandi, Taghdir, & Hosamirudsari, 2020; Lai, Shih, Ko, Tang, & Hsueh, 2020; Tian et al., 2020). There is, however, a growing and solidifying body of evidence reporting on cochleovestibular signs and symptoms, such as sudden sensorineural hearing loss (SSNHL), tinnitus and vertigo in adults with COVID-19, and refer findings on hearing screening in neonates whose mothers were COVID-19 infected (Fancello et al., 2021; Jacob, Flannery, & Mostert, 2020; Karimi-Galougahi, Naeini, Raad, Mikaniki, & Ghorbani, 2020; Koumpa, Forde, & Manjaly, 2020; Parrino et al., 2022). Jacob et al. (2020) reported on the novel ear, nose and throat triad of hearing loss, anosmia and ageusia, which brings a closer relationship of the commonly reported symptoms of COVID-19 and hearing loss to the fore. Munro, Uus, Almufarrij, Chaudhuri and Yioe (2020) maintained that more than 1 in 10 adults with COVID-19 complain about changes in their hearing status when interviewed 2 months post hospital discharge. This is while Parrino et al. (2022) reported on overall significantly higher incidence of SSNHL and combined acute cochleovestibular involvement during the pandemic than before.

If COVID-19 impacts cochleovestibular function in a similar manner to other viruses such as HIV, that is, either directly (virus itself) or indirectly (iatrogenic causes because of the medications prescribed to treat it) (Khoza-Shangase, 2020), implications for ear and hearing care in low- and middle-income countries (LMICs) such as South Africa are significant. This concern is driven by the numbers of infections that have and continue to be high in the African context – with minimal success from vaccination drives because of vaccine hesitancy (Cooper, Van Rooyen, & Wiysonge, 2021; Hughes, Mbamalu, Okonji, & Puoane, 2021). With the numbers of infection and the documented capacity versus demand challenges insofar as provision of ear and hearing care services is concerned in South Africa (Pillay, Tiwari, Kathard, & Chikte, 2020), it becomes crucial that clear definition of morbidity, including cochleovestibular symptoms, in order to facilitate proper planning at all levels of preventive audiology from primordial to tertiary (Khoza-Shangase, 2022).

This clear categorisation of symptomatology is important because of numerous reasons that are risk factors for cochleovestibular pathology and would impact ear and hearing care in the South African context, including (1) the unique circumstances presenting in South Africa when compared with China or various other regions such as North America and Europe in terms of quadruple burden of disease (Khoza-Shangase, 2021; Mukandavire et al., 2020; Pillay-van Wyk et al., 2020), with most of these cases not on treatment (Hansoti et al., 2019); (2) the risk factors for COVID-19 infection and its consequent mortality, for example, comorbid conditions such as hypertension, diabetes and chronic pulmonary disease are documented to be highly prevalent in South Africa (Chutel & Dahir, 2020); and (3) vaccine access and hesitancy (Cooper et al., 2021; Pepperrell et al., 2021) and poor healthcare infrastructure (Mezue et al., 2020; Ogbolosingha & Singh, 2020), as well as the prevailing poor social determinants of health (Ataguba & Ataguba, 2020; Khoza-Shangase & Mophosho, 2021). These factors highlight the need for extending the COVID-19 discussions from mortality-focused deliberations only to acute and long-term effects of COVID-19 as part of both prevention of the disease and efficient management of those infected (Kilic et al., 2020).

At the initial stages of the COVID-19 pandemic, understandably, significant focus was placed on life-threatening symptoms of the disease; however, with increasing numbers of infections and more evidence becoming available, new symptoms are being reported and scrutinised as the virus presents deleterious effects on particular organs and structures of the body. These innumerable other symptoms that are constantly documented indicate involvement of multiple systems by COVID-19 (Eravci et al., 2020; Savtale, Hippargekar, Bhise, & Kothule, 2021). With COVID-19 being a viral infection with direct cytopathic effects (Eravci et al., 2020; Kaur, Bherwani, Gulia, Vijay, & Kumar, 2020), which Munro et al. (2020) argued may contribute to hearing or balance disorders, the importance of this study is highlighted. Furthermore, as COVID-19 is a viral infection, prompt diagnosis of hearing impairment is critical because prompt treatment of viral-induced hearing loss has been proven to yield positive outcomes, while unidentified or late-identified permanent hearing impairment has well-documented negative impacts on the quality of life of the individuals affected (Saniasiaya, 2021). Interestingly, Jin et al. (2021) also reported that quality of life has been impacted by COVID-19 by raising mental-related disease patients of the otolaryngology department (e.g. panic, anxiety, depression and sleep disorders).

While other rapid and systematic reviews have been conducted on this topic (Almufarrij & Munro, 2021; Jafari, Kolb, & Mohajerani, 2021; Saniasiaya, 2021), albeit with different questions being explored, the nature of the pandemic, confirmatory studies such as this study are justified by COVID-19’s presentation and its management, as illustrated by (1) the rapid evolution of the virus along with new variants and subvariants; (2) the impact of individual and heard immunity developments; (3) the available COVID-19 treatments, including the different vaccines available to prevent it; and (4) the evolving nature of the clinical features from the initially reported to currently reported (Aleem & Slenker, 2021; Cascella, Rajnik, Aleem, Dulebohn, & Di Napoli, 2022; Wang et al., 2020; Zhou et al., 2020), among other things. In the context of a pandemic where the urgency of knowledge sharing is high, certain research and publication protocols that ensure research integrity may be relaxed (Fleming, Labriola, & Wittes, 2020; Park et al., 2021; Vindrola-Padros et al., 2020), thus replication of exploratory studies for confirmation of findings is arguably valuable, hence the importance of this study. Therefore, the purpose of this scoping review is to investigate published evidence, including case reports, on the impact of COVID-19 on the cochleovestibular system across the lifespan.

## Methodology

### Aim

This scoping review aimed to analyse published evidence on the impact of COVID-19 on the cochleovestibular system in line with the pandemic changes.

### Review approach

The Arksey and O’Malley’s (2005) framework for performing scoping reviews was utilised, as detailed next, with the guidelines of the Preferred Reporting Items for Systematic Reviews and Meta-analysis (PRISMA) followed during the screening process and for illustrating the process.

### Stage 1: Identification of the research question

The population (COVID-19 infected and/or exposed), concept (cochleovestibular symptoms or effects) and context (globally) (PCC) framework by the Joanna Briggs Institute was used to formulate the research question. For this review, the following question was formulated, ‘What evidence has been published on the impact of COVID-19 on the cochleovestibular system?’

### Stage 2: Data sources and search strategy

Levac, Colquhoun and O’Brien (2010) recommended that the search strategy should follow a transparent format that can be reviewed and it should be comprehensive, without compromising feasibility. Therefore, in this review, the literature search was conducted in December 2021 on databases including CINAHL, EBSCOHost, MEDLINE, ProQuest, PubMed, Scopus and ScienceDirect. The following terms and combination of keywords were devised and used for the search based on the research question and to ensure congruency between the research question, search terms and inclusion criteria. These terms included ‘COVID-19’ OR ‘SARS-CoV-2,’ OR ‘audiological effects of COVID-19’ OR ‘hearing loss and COVID-19’ OR ‘impact of COVID-19 on audiovestibular system’ OR ‘COVID-19 and cochleovestibular effects’, ‘auditory dysfunction’, ‘dizziness’, ‘hearing loss’, ‘vertigo’, ‘vestibular dysfunction’ and ‘otologic symptoms’. Ghalibaf et al. (2017) advised that utilising a combination of keywords enhances the search strategy to lead to an increased likelihood of finding more relevant articles while eliminates eliminating irrelevant articles.

### Inclusion criteria

To yield contemporary evidence, the search was restricted to peer-reviewed studies published in English during the COVID-19 era (between January 2020 and January 2022). To be part of the scoping review, the studies had to report on cochleovestibular symptoms or effects of COVID-19.

### Exclusion criteria

Review studies and studies not published in English were not eligible for inclusion. Studies were excluded if they did not address the research question, and if they did not include the combination of the search keywords. Studies that spoke about tinnitus on its own were excluded because of the limited objective evaluation of tinnitus in such studies and the multiple possible causal factors in the absence of hearing loss. Studies across the lifespan were included to ensure that findings assist with clinical planning across the lifespan, although it was obvious that identification of vestibular symptoms in the paediatric population would be challenging; however, hearing function findings would be valuable.

### Stage 3: Study selection

**FIGURE 1:**
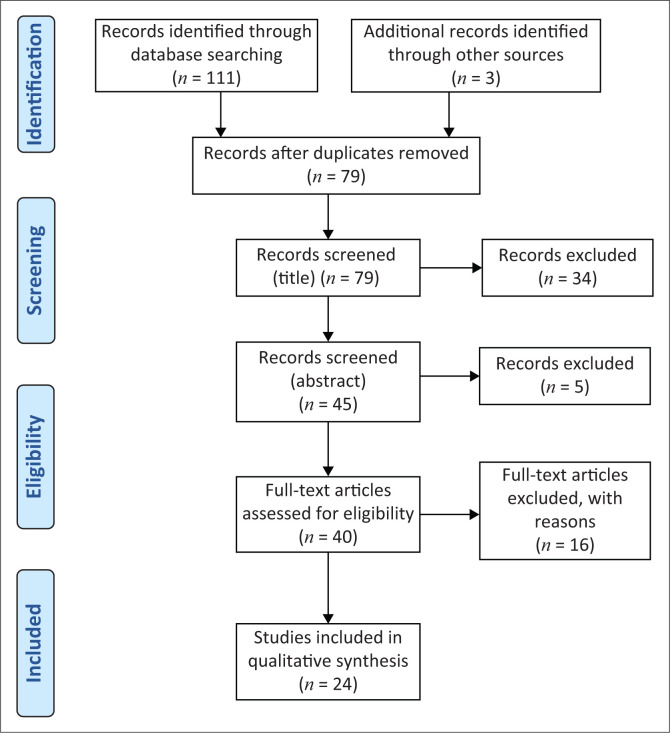
Preferred Reporting Items for Systematic Reviews and Meta-Analysis flow diagram for the current scoping review.

All in all, the database search from all databases identified 114 articles. All identified database citations were exported to a web-based bibliography manager, Endnote. Through this bibliography manager, duplicate studies were identified and removed. Following removal of duplicate studies, 79 studies remained, and these were brought down to 45 after title screening, where 34 articles were eliminated. The 45 abstracts were consequently screened, guided by the search question, and 5 studies were excluded because they did not report on cochleovestibular effects of COVID-19. A total of 40 studies were then assessed for eligibility, and of these, 15 were excluded because they did not meet the inclusion criteria. Ultimately, a full-text screening resulted in 24 studies meeting the inclusion criteria and being included in this scoping review. Figure 1 shows a PRISMA flowchart for the literature search, retrieval and inclusion process of this scoping review.

### Stage 4: Data extraction and charting

Following Peters et al.’s (2015) recommendation that the data must be charted in line with the objectives of the review, the charting was conducted. After the reference search, a total of 24 studies formed part of this review, and these are tabulated in Table 1. The table reflects the following data that were extracted from the included articles, with detailed analysis and discussion of the contents of the table presented next:

- researcher(s) and the year of publication
- title of the study
- country of publication
- aim of the article
- signs and symptoms reported
- conclusions
- recommendations.

**TABLE 1:** Summary of studies included in the scoping review documenting cochleovestibular impact of coronavirus disease 2019.

Manuscript number	Authors and date	Publication title	Context or country	Aim	Signs and symptoms	Conclusions	Recommendations
1	Karimi-Galougahi et al. (2020).	Vertigo and hearing loss during the COVID-19 pandemic: Is there an association?	Iran	Determining if there is an association between COVID-19 and vertigo and hearing loss	Acute-onset hearing loss and vertigo in six patients, with three presenting with positive polymerase chain reaction (PCR) findings.	Remains unknown if COVID-19 can invade the auditory and vestibular neural pathways, but it is possible. Urgent studies needed to examine any relationship between new onset otologic symptoms and COVID-19.	Prioritise performing screening PCR tests to determine a link, particularly in patients presenting with isolated balance disorder or hearing loss. Considerations around patient isolation and early and prompt therapeutic dilemmas such as treatment options such as empiric antivirals and the prescription of local or systemic steroids.
2	Koparal and Yılmazer (2021)	Evaluation of post-infection hearing with audiological tests in patients with COVID-19: A case-control study	Turkey	Evaluating the effects of COVID-19 infection on pure-tone average	Statistically significant differences at 4000 Hz, 6000 Hz and 8000 Hz between the two groups. Statistically significant differences in the right and left ears at 1000 Hz and 2000 Hz.	Significantly worse pure-tone average scores in the COVID-19 positive patients than in the healthy control group.	In cases presenting with unexplained hearing loss, COVID-19 should also be considered as a possible cause. Future studies should investigate the effects of COVID-19 on hearing and the pathophysiology underlying hearing loss in this population.
3	Jin et al. (2021).	Analysis of the characteristics of outpatient and emergency diseases in the department of otolaryngology during the ‘COVID-19’ pandemic	Shanghai, China	To evaluate changes in ordinary outpatient and emergency cases in otolaryngology during the COVID-19 pandemic	Different ranking of cases by diseases to previous ranking was found. Increased occurrence of conditions such as tinnitus, sudden deafness, chronic pharyngitis and allergic rhinitis, with decreased occurrence of acute laryngopharyngitis and acute pharyngitis.	Highlights the value of mental health observing, monitoring and maintaining.	Mental health should also be considered during this time.
4	Gallus et al. (2021).	Audiovestibular symptoms and sequelae in COVID-19 patients	Italy	Investigating general and audiovestibular features and sequelae in recovered patients and exploring for signs of residual or permanent hearing or vestibular loss	Hearing loss reported in 4 (8.3%) patients, tinnitus in 2 (4.2%), dizziness in 4 (8.3%), spinning in 1, vertigo (2%), dynamic imbalance 1 (2%) and static imbalance in 3 (6.3%). Resolution of audiovestibular symptoms in most cases post-COVID-19.	Audiovestibular symptoms mainly temporary in nature, when they do occur. Lack of clear evidence of persistent cochlear or vestibular damage post-COVID-19 recovery.	Further investigations are required.
5	Korkmaz, Eğilmez, Özçelik and Güven (2021).	Otolaryngological manifestations of hospitalised patients with confirmed COVID-19 infection	Turkey	Evaluating the incidence and characteristics of otolaryngology symptoms in COVID-19 patients	Hyposmia or anosmia (37.9%) and hypogeusia or ageusia (41.37%) were the most common otolaryngological conditions found. In descending order, the rate of otological or vestibular symptoms was dizziness (31.8%), tinnitus (11%), true vertigo (6%) and hearing loss (5.1%).	Otolaryngological manifestations occur in COVID-19 positive patients.	The COVID-19 should be considered in such cases, and proper investigations carried out.
6	Ricciardiello et al. (2021).	Sudden sensorineural hearing loss in mild COVID-19: Case series and analysis of the literature	Italy	Investigating the role of COVID-19 as a cause of sudden sensorineural hearing loss (SSNHL) Presenting five cases of SSNHL during COVID-19	The SSNHL did not present as a first symptom of COVID-19. Audiovestibular symptoms presented > 6 days after COVID-19 diagnosis, or post recovery from COVID-19. Both ears had equal chance of being affected and the loss could be either unilateral or bilateral. Most of the patients achieved partial recovery of auditory function, with complete recovery also found in one patient as indicated by the overall and individual patients’ mean hearing level graphical analyses. Except for 1 patient (no. 2), all reported contextual onset of tinnitus. All cases’ tinnitus significantly improved post therapy.	The SSNHL could be an infrequent feature of COVID-19, even in cases where COVID-19 disease manifestation is mild.	Highlight the significance of early detection and management of SSNHL and other occasional symptoms, facilitating early treatment.
7	Beckers, Chouvel, Cassetto and Mustin (2021)	Sudden sensorineural hearing loss in COVID-19: A case report and literature review	Belgium	Presenting the 9th case of SSNHL in a SARS-CoV-2- positive patient	Clinical and technical assessment indicated cophosis on the right side with normal tympanometry and otomicroscopy. A deficit of the right anterior semi-circular canal was found on Video Head Impulse Testing. Audiology: auditory brainstem responses (ABR) and pure tone measures revealed severe-profound sensorineural hearing loss	Case report contributes towards documented evidence on links between hearing loss and COVID-19.	Inclusion of PCR testing in the diagnostic workup of patients with SSNHL during COVID-19.
8	Pokharel, Tamang, Pokharel and Mahaseth (2021)	Sudden sensorineural hearing loss in a post-COVID-19 patient	Nepal	Presenting a case of an apparently healthy 27-year-old male with SSNHL without any known ear pathology or comorbidities	Normal outer and middle ear. Moderately severe left SSNHL with normal hearing in the contralateral ear. Significant improvement in hearing function following treatment with oral steroids.	Direct links to COVID-19 as cause of hearing loss speculated. No clear pathophysiologic mechanism found that links SSNHL to COVID-19.	Comprehensive case history and diagnostic assessment be conducted in SSNHL cases with recent or past COVID-19 infection because SSNHL could be a consequence of COVID-19.
9	Kilic et al. (2020)	Could sudden sensorineural hearing loss be the sole manifestation of COVID-19? An investigation into SARS-COV-2 in the aetiology of sudden sensorineural hearing loss	Turkey	Investigating the presence of COVID-19 in patients with only SSNHL during COVID-19	Five male patients unilateral SSNHL as a sole complaint. RT-PCR testing for COVID-19 was positive in one of the five cases and negative in the rest.	The SSNHL could be the only sign of COVID-19 infection.	Raised awareness of this phenomenon is important during the COVID-19 pandemic so as to prevent the infectious spread through adhering to regulations such as isolation and institution of early COVID-19 targeted treatment.
10	Chern, Famuyide, Moonis and Lalwani (2021)	Bilateral sudden sensorineural hearing loss and intralabyrinthine haemorrhage in a patient with COVID-19	United States of America	Describing a case of bilateral SSNHL and intralabyrinthine haemorrhage in a patient with COVID-19	Bilateral moderate-severe SSNHL, vertigo and bilateral aural fullness in an adult woman with COVID-19. Intralabyrinthine haemorrhage bilaterally (worse in left). Vestibular symptoms near-resolution, with a fluctuating right SNHL and a severe-profound left mixed hearing loss post- treatment.	Otologic symptoms possibly because of intralabyrinthine haemorrhage caused by direct viral invasion of the labyrinth or cochlear nerve and COVID-19-associated coagulopathy.	Consider COVID-19 as a possible cause.
11	Kimura et al. (2021)	The COVID-19 findings revealed via otolaryngological examination: Findings of a Japan Otorhinolaryngologist Association questionnaire	Japan	Analysing the characteristics of otorhinolaryngological findings to improve COVID-19 diagnostic systems in a primary care setting	Of the 350 cases considered in this study, hearing impairment was found in 2 (1%) and otalgia in 2 (1%).	Challenging to differentiate presenting symptoms from those of bacterial infections.	No conclusive published links to COVID-19 established.
12	Alan and Alan (2021)	Hearing screening outcomes in neonates of SARS-CoV-2 positive pregnant women	Turkey	Investigating potential links between maternal COVID-19 and newborn hearing loss	Neonates in the COVID-19 group more likely to present with a ‘refer’ finding in ABR when compared with the control group. Repeat ABR findings essentially similar between the groups. Positive PCR findings during the second trimester more likely to lead to the initial ‘refer’ ABR result.	Positive COVID-19 PCR results during pregnancy are significantly linked with a higher risk of abnormal newborn hearing screening (NHS) outcomes. Abnormal NHS findings may be associated with the timing of positive PCR results during pregnancy (trimester).	Maternal COVID-19 infection is a possible risk factor for hearing loss in infants in this population, even if the hearing loss in temporary. The COVID-19 induced delayed neuromaturation because of factors such as preterm delivery, foetal growth restriction, and perinatal mortality possibly raises the ‘refer’ rate. Additionally, high levels of maternal inflammation as a response to viral infection can affect a few aspects of the foetus’ developing brain, causing numerous diverse neurological consequences.
13	Munro et al. (2020)	Persistent self-reported changes in hearing and tinnitus in post-hospitalisation COVID-19 cases	UK	Reporting on hearing changes in 138 adults following hospitalisation for COVID-19	Hearing changes and tinnitus was reported by 16 (13.2%) patients since COVID-19 diagnosis. One patient with hearing loss also presented with vertigo. One patient reported unilateral left tinnitus with a sensation of aural pressure. Another patient reported tinnitus symptom resolution.	Because audiological testing was not conducted, results were not conclusive as various confounding factors such as surrounding environmental changes because of hospitalisation, use of face masks and ototoxic medications as part of critical care, etc., are potential explanations for changes in subjective hearing and sudden awareness of pre-existing hearing loss and tinnitus. Other possible causes should be considered for example, auto-immune diseases, vascular disorders, etc.	High-quality studies to examine the acute and temporary effects, as well as the longstanding risks of COVID-19 on the audiovestibular system are required. Longitudinal assessments of COVID-19 patients crucial for determination of long-term sequelae.
14	Parrino et al. (2022)	Sudden hearing loss and vestibular disorders during and before COVID-19 pandemic: An audiology tertiary referral centre experience	Italy	Evaluating the impact of the COVID-19 pandemic on the incidence of acute hearing and vestibular disorders	Increased annual incidence of total acute audiovestibular disorders during COVID-19, but no statistically significant differences when compared with pre-non-COVID-19 years. Significantly higher incidence of SSNHL and combined acute cochleovestibular involvement during COVID-19 period when compared with previously.	The SSNHL during COVID-19 seemed worse using pure-tone average, with an increased incidence of associated vestibular involvement.	Further studies are required to clearly establish the relationship between COVID-19 and audiovestibular disorders incidence and pathophysiology.
15	Degen, Lenarz and Willenborg (2020)	Acute profound sensorineural hearing loss after COVID-19 pneumonia	Germany	Presenting a 60-year-old man with COVID-19 and reported deafness with a loud tinnitus (white noise) bilaterally after recovery	Complete right deafness and profound left SNHL from audiologic testing. Signs of an inflammatory process in the cochlea from MRI findings. Cochlear implant performed urgently under local anaesthesia.	An immune-mediated inflammation could be triggered by COVID-19 because the virus, in severe cases, has been linked to a dysregulation of the immune system.	The value of prompt radiologic and audiologic assessments in COVID-19 patients with sudden hearing loss highlighted, particularly if the patient also presents with neurologic symptoms.
16	Mustafa (2020)	Audiological profile of asymptomatic COVID-19 PCR-positive cases	Egypt	Comparing the amplitude of transient evoked otoacoustic emissions (TEOAEs) and pure tone audiometry thresholds between asymptomatic COVID-19 positive cases and normal noninfected individuals	Significantly worse high-frequency pure-tone thresholds and TEOAE amplitudes in the COVID-19 positive group.	Possibility of COVID-19 infection having adverse effects on cochlear hair cell function even where patients are asymptomatic established. Lack of major COVID-19 symptoms does not mean that the cochlea is healthy and intact.	Further research is required to establish the mechanism of the COVID-19 cochlear effects.
17	Yıldız et al. (2021).	Hearing test results of newborns born from the coronavirus disease 2019 (COVID-19) infected mothers: A tertiary centre experience in Turkey	Turkey	Establishing whether COVID-19 infection during pregnancy can cause congenital hearing loss	Of the newborns sample, 10.5% presented with unilateral hearing loss on initial testing. On repeat testing 15 days later, all newborns in the sample presented with normal hearing.	The lack of hearing loss in the current sample does not preclude COVID-19 as a possible cause of congenital hearing loss.	Larger patient series should be investigated for more conclusive findings. Late onset hearing loss should be kept in mind in this population, therefore longitudinal follow-up important.
18	Koumpa et al. (2020).	Sudden irreversible hearing loss post- COVID-19	UK	Presenting the first case of SSNHL following COVID-19	Unilateral left severe-profound high frequency SNHL with tinnitus post 1 week hospitalisation and treatment for COVID-19. Slight improvement in symptoms after intratympanic steroid administration.	Consider COVID-19 in sudden SNHL cases during the pandemic.	Raised awareness and standard screening for SSNHL following COVID-19 infection facilitates prompt prescription of steroids, which enables better outcomes in terms of recovering hearing.
19	Oskovi-Kaplan et al. (2022).	Newborn hearing screening results of infants born to mothers who had COVID-19 disease during pregnancy: A retrospective cohort study	Turkey	Investigating the incidence of hearing loss in infants born to mothers who had COVID-19 infection during pregnancy, through neonatal hearing screening	No significant difference in hearing screening results between the two groups at final screening.	No evidence of COVID-19 infection during pregnancy being a risk factor for neonatal hearing loss.	Large-scale, multicentre studies are required to confirm or dispute current findings regarding neonatal outcomes.
20	Bhatta et al. (2021).	Study of hearing status in COVID-19 patients: A multicentred review	Nepal and India	Evaluating the hearing status of COVID-19 patients and comparing that with a control group, using pure tone and impedance audiometry	All aural symptoms present at initial assessment resolved at 3 month follow-up. Symptoms included tinnitus in 1.8%, aural fullness in 1.4%, hearing loss in 3.9% and earache in 1.8% of the sample at initial testing.	No significant difference in the hearing status of the COVID-19 positive patients when compared with the control group.	Future studies required.
21	Dharmarajan et al. (2021).	Hearing loss: A camouflaged manifestation of COVID-19 infection.	India	Assessing the audiological profile of 100 mild to moderately affected COVID-19 individuals to establish otologic manifestations of COVID-19	High frequency hearing loss and referred OAE findings, with SNHL being the most prevalent type of hearing loss. Six patients presented with conductive hearing loss.	To maintain and improve quality of life of affected individuals, early identification and intervention is important.	-
22	Savtale et al. (2021).	Prevalence of otorhinolaryngological symptoms in COVID-19 patients	India	Determining the prevalence of ENT symptoms in COVID-19 positive patients	In a sample of 180 patients, 112 presented with one or more ENT symptoms that included throat pain (47.2%), loss of smell (55.5%), loss of taste (58.8%) and hearing loss and tinnitus (54.44%) – with generalised COVID-19 symptoms. Hearing loss was sudden and more prominent in the older age group. A total of 81.6% of those who reported hearing loss reported complete symptom resolution within 8 ± 2 days.	The ENT symptoms can be regarded as cardinal features for early diagnosis of COVID-19, thus facilitating prompt intervention and isolation for infection spread control.	Coronavirus disease 2019 should be considered a possible cause and thus proper investigations should be performed so that appropriate management can be instituted.
23	Sriwijitalai and Wiwanitkit (2020).	Hearing loss and COVID-19: A note	Thailand	A case note	One (first reported case) of an elderly female COVID-19 patient with unilateral SNHL.	Possibility of COVID-19 causing hearing loss raised.	-
24	Rhman and Wahid (2020).	The COVID-19 and sudden sensorineural hearing loss: A case report	Egypt	Case report of a 52-year-old man with sudden onset left hearing loss, following progressive tinnitus. No otalgia, otorrhea, dizziness or vertigo	Unilateral hearing loss – left severe SNHL. Degree of hearing loss improved by 20 dB – 30 dB following intra-tympanic injection of corticosteroid.	The SSNHL can be the sole presenting symptom during COVID-19 infection.	Considerations around intra-tympanic corticosteroid injection versus use of systemic steroids in the treatment of COVID-19 patients. The COVID-19 should be taken into consideration in patients presenting with sudden hearing loss in this era. Further research is required to establish pathogenesis and auditory complications of COVID-19.

Data analysis was carried out through descriptive analysis, as the last step set out by Arksey and O’Malley (2005), with implications of the findings focusing on three broad categories: implications for teaching, research and practice – highlighting planning needs (Peters et al., 2015).

### Ethical considerations

This scoping review followed all ethical standards for a study that does not involve direct contact with human or animal participants, including reflexivity and informed subjectivity, audience-appropriate transparency and purposefully informed selective inclusivity (Suri, 2020).

## Results and discussion

The characteristics and main cochleovestibular findings documented in the 24 studies that met the eligibility criteria are depicted in Table 1. These articles varied in terms of type of study, including case reports, single group prospective, case-control prospective, retrospective or cross-sectional studies on the cochleovestibular impact of COVID-19. The articles are from both LMICs and high-income countries (HICs), with no evidence from Africa as a continent, except for some from transcontinental Egypt (Mustafa, 2020; Rhman & Wahid, 2020). The majority of the evidence was on adults, with minimal but emerging evidence from the paediatric population (Alan & Alan, 2021; Oskovi-Kaplan et al., 2022; Yıldız et al., 2021). The current findings are valuable for the South African context as they seem to flag a need for preventive ear and hearing measures planning, because they indicate the possibility of cochleovestibular presentation in COVID-19. Furthermore, current findings bring forward significant implications not only for therapeutic interventions insofar as prompt otorhinolaryngological management is concerned but they also highlight such implications for patient isolation should cochleovestibular symptoms be the first and only symptom, as well as for audiological service provision and human resource planning within this resource-constrained context.

As far as the cochleovestibular symptoms are concerned, there is no conclusive published evidence of cochlear or vestibular damage that is persistent and clinically relevant following COVID-19 recovery (Gallus et al., 2021). However, the current review appears to indicate a strong possibility of COVID-19 impacting cochleovestibular function in various ways across the lifespan (Alan & Alan, 2021; Gallus et al., 2021; Korkmaz et al., 2021); however, this evidence is not strong, and it remains difficult to confirm because of two key factors: (1) poor quality of studies and evidence that is based mainly on case studies (Almufarrij, Uus, & Munro, 2020; Munro et al., 2020), and (2) occurrence of other possible causes of cochleovestibular symptoms in these patients (Munro et al., 2020). Although in the current scoping review, the quality of the studies was not formally assessed, a scoping overview indicates the following features of the studies: (1) mostly case reports where other possible causes are a possibility; (2) limited use of objective audiological measures, but rather reports and complaints by patients were used; (3) where control groups are included, there is limited clarity provided on inclusion criteria for the control group participants, and so on. These limitations are, however, arguably expected during a new public health emergency where everything is new and novel and exploratory research is what is feasible.

Nonetheless, notwithstanding this research quality challenge, the limited evidence that exists seems to warrant the need for careful audiological assessment and monitoring and polymerase chain reaction (PCR) testing for COVID-19 diagnosis in patients with sudden unexplained cochleovestibular features during COVID-19 (Beckers et al., 2021; Jafari et al., 2021; Koparal & Yılmazer, 2021) and children born to mothers who were COVID-19 positive during pregnancy (Alan & Alan, 2021; Oskovi-Kaplan et al., 2022; Yıldız et al., 2021). This is particularly important as definitive evidence is required to ensure that appropriate treatment is provided that takes into cognisance the therapeutic dilemmas surrounding the use of steroids in this population, as raised by Karimi-Galougahi et al. (2020), the need for patient isolation and the possibilities of monitoring for late onset hearing loss in children born to mothers who were COVID-19 positive during pregnancy. The South African healthcare system and the audiology community would need to carefully plan around how to effectively manage these cases within the already resource-constrained context (Pillay et al., 2020), possibly within already existing programmes, adopting a programmatic approach to care (Khoza-Shangase, 2022).

Analysis in this review leads the current author to conclude that generally, cochleovestibular symptoms that have been reported in adults include sudden onset sensorineural hearing loss with varying severity levels, which can be unilateral or bilateral, mostly high-frequency configuration, with or without tinnitus and vertigo, with the presentation in the paediatric population remaining relatively obscure. The occurrence of cochleovestibular symptoms is reported to vary, with some patients presenting with SSNHL as a leading symptom of COVID-19, while in others the symptoms occur greater than 6 days after the COVID-19 diagnosis or long after recovery from the infection (Kilic et al., 2020; Munro et al., 2020; Pokharel et al., 2021; Ricciardiello et al., 2021). Even if some patients present with cochleovestibular symptoms, some evidence seems to indicate that these are mainly temporary in nature (Gallus et al., 2021; Munro et al., 2020). In paediatrics, *refer* findings have been documented during neonatal or newborn hearing screening (NHS) in neonates whose mothers were infected with COVID-19, particularly in the second trimester. In most of the cases, the symptoms are temporary and resolve either partially or completely following treatment with steroids, thus highlighting the importance of clear categorisation and identification of these symptoms for early intervention. The possibility of late onset of hearing loss in the paediatric population has not been eliminated by current data and warrants closer monitoring of these patients, thus raising implications for longitudinal follow-up of these children for early intervention to be instituted should the need arise, within the South African context.

The type of onset of the cochleovestibular symptoms is reported to be acute (Gallus et al., 2021; Koumpa et al., 2020; Savtale et al., 2021), with most of the patients reported to have achieved partial or complete resolution of symptoms after therapy with steroids (Bhatta et al., 2021; Chern et al., 2021; Koumpa et al., 2020; Ricciardiello et al., 2021). For example, Pokharel et al. (2021) reported on significant improvement in hearing following treatment with steroids. Early detection and diagnosis of SSNHL and other less familiar symptoms in this population provides patients with the prospect to receive early intervention (Koumpa et al., 2020; Ricciardiello et al., 2021). Koumpa et al. (2020) argued that this prompt screening and diagnosis of cochleovestibular symptoms post-COVID-19 facilitates prescription of an early course of steroids, thus providing the patient with the best chance of symptom resolution. Koparal and Yılmazer (2021) stressed that in cases presenting with unexplained hearing loss or vestibular dysfunction, it is critical to prioritise PCR testing to determine the possibility of COVID-19 causing the symptoms because of two important reasons: (1) prevention of virus spread (patient isolation) and (2) offering the patient therapy for acute hearing loss with steroids while taking into cognisance the therapeutic dilemmas surrounding the use of steroids in this population (Karimi-Galougahi et al., 2020), thus affording the infected patient an opportunity to recover from the symptoms.

From their review, Almufarrij et al. (2020) concluded that significantly few cochleovestibular symptoms can be confirmed to be COVID-19 related, with most of the cases presenting with minor symptoms. Mustafa (2020), however, highlighted that the lack of significant COVID-19 symptoms does not necessarily provide assurance that cochlear function is healthy and safe. Several studies reviewed indicate the presence of SSNHL with or without tinnitus and vertigo (Beckers et al., 2021; Chern et al., 2021; Gallus et al., 2021; Jin et al., 2021; Kimura et al., 2021; Korkmaz et al., 2021; Koumpa et al., 2020; Munro et al., 2020; Ricciardiello et al., 2021) with nonspecific symptoms such as SSNHL being reported as possibly being the sole feature that could be used to identify a COVID-19 case (Kilic et al., 2020). The reported rate of otological or vestibular symptoms varied significantly. For example, Korkmaz et al. (2021) reported the rate of hearing impairment (5.1%), tinnitus (11%), dizziness (31.8%) and true vertigo (6%); Gallus et al. (2021) documented their rate at 8.3% for hearing loss, 4.2% for tinnitus and 8.3% for dizziness; Bhatta et al. (2021) reported hearing loss in 3.9%, tinnitus in 1.8%, earache in 1.8% and aural fullness in 1.4% of their patients with COVID-19; and Munro et al. (2020) reported a change in hearing and tinnitus in 13.2% of their COVID-19 diagnosed patients. Munro et al. (2020) reported that testing showed vestibular symptoms, with Beckers et al. (2021) reporting a deficit of the anterior semicircular canals and Chern et al. (2021) documenting bilateral intralabyrinthine haemorrhage.

Ricciardiello et al. (2021) reported that both ears can equally be affected, and the impairment could be unilateral or bilateral, and if bilateral, the symptoms can be symmetrical or nonsymmetrical (Karimi-Galougahi et al., 2020; Koparal & Yılmazer, 2021; Koumpa et al., 2020; Sriwijitalai & Wiwanitkit, 2020), with the severity of the hearing loss documented to range from mild loss to profound hearing loss (Beckers et al., 2021; Chern et al., 2021; Koparal & Yılmazer, 2021; Koumpa et al., 2020; Pokharel et al., 2021) and some evidence of pure-tone average being substantially worse in patients with COVID-19 when compared with healthy control groups, particularly in the high frequencies (Koparal & Yılmazer, 2021). Mustafa (2020) found that pure-tone hearing thresholds in high frequencies and amplitudes of transient evoked otoacoustic emissions (TEOAE) were significantly worse in this population, thus concluding that COVID-19 infection potentially has adverse effects on cochlear hair cell function even when infected individuals are asymptomatic for the cardinal disease symptoms. Similar findings were reported by Dharmarajan et al. (2021), while Daher et al. (2022) only found the hearing loss in the ultra-high frequencies, thus highlighting the need for inclusion of sensitive audiological measures such as ultra-high frequency audiometry and distortion product OAEs (DPOAEs).

Among the paediatric population, COVID-19 positivity in pregnancy has been associated with an increased risk of *refer* screening results during NHS (Alan & Alan, 2021; Oskovi-Kaplan et al., 2022; Yıldız et al., 2021). Alan and Alan (2021) reported that PCR positivity during the second trimester of pregnancy may be associated with abnormal NHS findings, although these *refer* findings resolve on repeat screening later and results become similar to those of the control group (Alan & Alan, 2021; Yıldız et al., 2021; Oskovi-Kaplan et al., 2022). In fact, Oskovi-Kaplan et al. (2022), based on the lack of differences they found between the study and control groups at final screening, conclude that COVID-19 infection during pregnancy does not appear to be a risk factor for hearing loss – a sentiment not shared by Alan and Alan (2021), who asserted that maternal COVID-19 infection could potentially be a risk factor for hearing impairment, albeit the effect being temporary in nature. On the other hand, Yıldız et al. (2021) suggested that for conclusive findings about hearing loss because of COVID-19 in this population, larger patient series in newborns should be evaluated and that late onset hearing loss is also a possibility in this population. Oskovi-Kaplan et al. (2022) supports this suggestion by recommending that large-scale, multicentre studies of pregnant women be conducted to support current evidence and to make a definitive judgment regarding neonatal hearing outcomes post-COVID-19-exposure and infection. This raises research and clinical follow-up implications for the South African research and audiology communities.

Findings from the review reveal uncertainty and inconsistency regarding the underlying pathophysiology of the cochleovestibular symptoms in COVID-19 positive patients, with Pokharel et al. (2021) maintaining that the absence of a clear pathophysiologic mechanism linking COVID-19 to SSNHL creates a vague association. Therefore, Koparal and Yılmazer’s (2021) recommended more research to examine the impact of COVID-19 on hearing, including the underlying pathophysiology. So far, current evidence suggests that otologic features found in this population are potentially because of (1) direct viral invasion of the labyrinth or cochlear nerve and intralabyrinthine haemorrhage from COVID-19-associated coagulopathy (Chern et al., 2021); (2) an inflammatory process in the cochlea (virus-triggered, immune-mediated inflammation), as observed in MRI findings (Degen et al., 2020); (3) the possibility of COVID-19 invading the neural pathways involved in hearing and balance (Karimi-Galougahi et al., 2020); and (4) in neonates, delayed neuromaturation because of factors such as preterm birth, foetal growth restriction and perinatal mortality (Alan & Alan, 2021). On the other hand, Kimura et al. (2021) argued that differentiating symptoms in COVID-19 from those of bacterial infections is difficult, while Munro et al. (2020) highlighted that many other potential causes of hearing loss linked to critical care such as auto-immune disease, systematic or local infections, ototoxic medications and vascular disorders should also be considered.

Numerous recommendations were found in the studies reviewed regarding establishment and assessment and management of cochleovestibular symptoms in patients with COVID-19. Key to the recommendations is one by Karimi-Galougahi et al. (2020) and Parrino et al. (2022), who argue that epidemiological correlations and bio-pathological mechanisms involved in cochleovestibular presentation of COVID-19 require further investigations. Linked to this recommendation, is ensuring that studies should not be of poor quality (Almufarrij et al., 2020; Fancello et al., 2021; Munro et al., 2020), with Almufarrij et al. (2020) suggesting that future high-quality studies be conducted once the life-threatening nature of the pandemic is under control. Such high-quality studies would ensure that the distinction between premorbid and current changes in hearing, balance and tinnitus is clear and that the possibility that environmental surroundings changes associated with use of masks and hospitalisation is not what may have caused recognition of pre-existing tinnitus and hearing loss (Munro et al., 2020). Jafari et al. (2021) suggested future directions including follow-up assessments utilising reliable objective measures, as well as studies with properly constituted research designs.

Fancello et al. (2021) believed that despite the fact that the quality of the current evidence is poor, there is sufficient support for cochleovestibular disorders to be included as possible important features of COVID-19. Such heightened awareness of cochleovestibular disorders as part of the nonspecific presentation of COVID-19 is important, as it facilitates prevention of the spread of this infectious pandemic through patient isolation and provision of and early COVID-19 specific interventions (Kilic et al., 2020; Koumpa et al., 2020). Pokharel et al. (2021) recommended a comprehensive history and diagnostic assessment for past or recent COVID-19 infection in individuals with SSNHL, because SSNHL could be a sequela of COVID-19 (Ricciardiello et al., 2021), with Beckers et al. (2021) recommending inclusion of PCR testing in the diagnostic evaluation of these patients with SSNHL during COVID-19. Furthermore, Munro et al. (2020) recommended meticulous following up of COVID-19 patients for establishment of its long-term consequences on cochleovestibular function.

## Conclusion

The current scoping review aimed at answering the question ‘what evidence has been published on the impact of COVID-19 on the cochleovestibular system?’ revealed findings that highlight a need for high-quality research in the area so that definitive answers can be provided. Current evidence suggesting the possibility of cochleovestibular impact of COVID-19 across the lifespan is not strong enough; however, it does show that tinnitus, vertigo and SSNHL of mostly high frequencies occur either singly or in combination in adults and *refer* findings in neonatal hearing screening, albeit temporary. These cochleovestibular symptoms occur sometimes as the only symptom of COVID-19 in the absence of the standardly reported core symptoms, and these are generally temporary and resolve either partially or completely following treatment with steroids. The pathophysiologic mechanism for the cochleovestibular symptoms in this population remains obscure. This lack of clarity and definitiveness is the same in the adult and paediatric populations, thus raising a need for caution around assessment and management of patients presenting with unexplained cochleovestibular symptoms during this pandemic and a need for possible longitudinal monitoring of children born to mothers who were COVID-19 positive during pregnancy. Recommendations for PCR testing have been made to allow for accurate and safe treatment of these patients – including isolation should PCR come back positive for COVID-19, with safety and possible value around the use of steroids for treatment of SSNHL in this population being highlighted. These findings have important implications for clinical care planning and evidence development within the South African context, as detailed in the results and discussion section of this article. Considerations around preventive approaches from primordial to tertiary prevention levels are crucial for this context, with implications for resource allocation taken careful cognisance of.
